# The Edinburgh Medical and Surgical Journal

**Published:** 1834-07-01

**Authors:** 


*The Edinburgh Medical and Surgical Journal, Nos.* CXVIII. *and* CXIX. *January and April,* 1834.

We return with pleasure to the pages of our able contemporary; for we feel assured that few things can be more instructive or more interesting than original papers, when condensation has added brevity to their other merits.

Dr. Wm. Turnbull has given his second *Medico-Chirurgical Report of the Huddersfield Infirmary,* from which we made an extract in the Collectanea of our last number. After narrating some cases of cholera, which seem to favour the theory of the contagionists, Dr. Turnbull gives the following account of the ravages committed by scarlatina in 1832.

"On the cessation of cholera, scarlet fever made its appearance. One of the first cases I met with was one of Scarlatina anginosa, in a little boy of my own, which was ushered in by convulsions. It occurred in the end of September. Subsequently the disease became very general, and in its most malignant form. Great numbers of children, and some adults, were cut off, more especially to the south of Huddersfield, in and near Almondbury. Delirium set in almost from the first; the heat was irregularly distributed; the eruption indistinct; the pulse rapid and feeble; and the throat speedily became gangrenous. Generally, also, the neck was greatly swollen. In the majority of the fatal cases death happened on the fifth day, in not a few on the fourth, and in some still earlier. I knew of one instance of a servant girl, eighteen years of age, in a family which had previously lost by the malady one of its members, to whom the girl was much attached, who began to complain on the Sunday evening, had a slight show of the eruption on the Monday, and died early on the Tuesday morning; the duration of her illness having been less than thirty-six hours.

"When the disease commenced with such intensity, remedial means proved of little avail. In some cases, however, ammonia, quinine, wine, and the warm bath, appeared to be beneficial, and to abate the extreme restlessness which characterized most of the cases; but, on the whole, the results of treatment were far from satisfactory. November was the most fatal month, and onwards to January many continued to sink." (P. 5.)

The whole report does the author great credit. Were such accounts of hospital practice common, we might prophesy a speedy progress of the art of medicine.

Mr. MARSHALL has a sensible paper on *the Abuse of Spirituous Liquors among the Troops in India.* We deem it needless to quote from it, as the absurdity of making arrack a part of the soldier's rations is too obvious to need any comment.

Mr. POOLE gives an account of an *Epidemic Gastric Fever which prevailed in the Limerick Garrison during the Months of May, June, and July,* 1833.

"Diarrhœa invariably ushered in the complaint, and frequently reappeared during its course; but the bowels were at other times slow, and required to be unloaded. Every medicine in this way, however, required to be of the mildest nature, as even the gentlest laxative produced often violent effects. The fecal discharges were varied; in some they appeared wholly serous or mucous; in others natural, but liquid. Various intermixtures of colouring matter were frequent: in some blood, in others bile, and, in a case of the 83d regiment, what might be termed melæna. In this case large quantities of calomel and Cayenne pepper had been given by a practitioner in the country, who, fortunately for the patient, considered his case bad enough to have him removed to headquarters. Griping was a frequent attendant, but tenesmus was not observed. Vomiting of bilious-looking matter occurred in one case; but the stomach was generally retentive, though nausea was by no means unfrequent. Complete anorexia existed for many days. The patients obstinately refused to eat anything, appearing to loath the sight of food, or the idea of using it." (P. 36.)

The treatment employed consisted chiefly in the use of leeches, counter-irritants, hyoscyamus, and the acetate of morphia. Every patient recovered.

There are some valuable observations in Dr. CHURCHILL's *Report of Diseases occurring at the Wellesley Dispensary for Lying-in Women, and Diseases of Females, during the Year* 1832. Dr. C. thinks the nitrate of silver injection the best remedy we possess in vaginal leucorrhœa, but he says that in uterine leucorrhœa astringent injections almost invariably aggravate the disease. The following practical remarks are well worthy of attention:

"In several patients who suffered much, the dysmenorrhœa proved no bar to conception, contrary to the general statements of authors.

"The first object in the treatment of amenorrhœa was, of course, to produce or recall the discharge: in some we were successful, in others we failed. In addition to the cases related by Dr. Bardsley, of Manchester, of the beneficial effects of strychnine, I have to record one to whom I gave it in doses of one twelfth of a grain, increasing to one eighth three times a day. In the course of a week the discharge appeared, having been absent ten months previously. It has since recurred naturally. In most cases there were symptoms present which precluded the use of this remedy. We tried aloes alone, and in combination with assafœtida, very extensively, and with great benefit. The menses often returned under its exhibition; and, when they did not, the general health was improved.

"The various preparations of iron were administered, and often with good effect.

"Some cases, however, resisted every remedy that was tried.

"The dysmenorrhœa was much relieved by the use of opium in grain-doses an hour or two before the expected attack, and during the paroxysm. The addition of two or three grains of camphor is often useful.

"I found opium in large doses an excellent medicine in hemorrhagia. It often stops, or at least diminishes the discharge, and is not attended with any inconvenience. Preparations of iron were of use, and frequently a blister to the loins." (P. 45.)

The next paper, which is by Dr. JAMES HENRY, is a curious instance of the varied lights which a man of talent can throw on the smallest subject; and, though we are far from admitting all the author's theories, we cannot but admire the ingenuity which dictated them. Dr. Henry administers sulphate of magnesia, with a large addition of sulphuric acid. His directions are as follows:

"Saturate any quantity of cold water with sulphate of magnesia; filter through paper, and add to every seven ounces of the solution one ounce of the dilute sulphuric acid of the Dublin or Edinburgh Pharmacopœias.

"Dose: one table-spoonful in a wine-glass of water.*

"In those cases in which the bowels are very easily moved, a single table-spoonful is sufficient to produce a considerable purgative effect.

"In ordinary cases, a table-spoonful, taken an hour or two before breakfast, produces one or two evacuations immediately after breakfast.

"In other cases the dose is to be repeated once or twice, at intervals of two or three hours, according to circumstances.

"Where the symptoms are urgent, a table-spoonful may be

* "Each table-spoonful contains about two drachms of sulphate of magnesia, and half a drachm of dilute sulphuric acid."

given every hour until the effect is produced; and, where the urgency is extreme, a saturated solution of the salt, containing only one half of the above-mentioned quantity of acid, may be given in doses of two table-spoonfuls, repeated every hour." (P. 48.)

Our readers will recollect, that the dilute sulphuric acid of the Dublin and Edinburgh Pharmacopœias is stronger than the London formula of the same name, in the proportion of four to three. Each dose, therefore, of Dr. Henry's favourite aperient contains *m.* xl. of the Acid. Sulph. dil. of the London Pharmacopœia; and this enormous quantity must, in most cases, be doubled or tripled before the bowels can be effectually moved. Surely a man may wish to give or take a decent dose of some efficient purgative, without desiring at the same time to acidify or be acidified at this rate. Then there is another obvious inconvenience in introducing sulphate of magnesia to such bad company: "The frequently-repeated contact of the acid saline solution being injurious to the teeth, it is useful to adopt the precaution of taking the medicine through a quill, or from the spout of a small teapot, whenever it is necessary to continue its use for any length of time." (P. 50, *note*.) Dr. Henry, however, nothing daunted, sees sixteen advantages in this method of giving Epsom salts, but we must be satisfied with citing seven.

"1. It is an effectual purgative, never failing to move the bowels in all cases in which the bowels can be moved by medicine. I am not acquainted with any purgative which is more certainly effectual.

"2. It is quick in its operation; the effect being produced in ordinary cases within two or three hours after the first or second dose, and a necessity rarely arising for the continuance of the medicine beyond the third dose.

"3. It is safe, never purging so as to produce exhaustion.

"4. It does not give rise to the slightest degree of nausea, but, on the contrary,

"5. Quickly puts a stop to nausea, and appeases irritability of the stomach.

"6. Flatulence, that most distressing attendant upon constipated bowels, is immediately and signally relieved by this medicine, which not only promotes the expulsion of the flatus already generated, but diminishes the tendency to its further secretion.

"7. In a few minutes after this medicine has been swallowed, so agreeable a sensation of warmth is felt in the stomach, that the medicine is not only readily taken, but even relished, by many persons whose stomach will not retain any other liquid purgative, unless impregnated with the hottest aromatic tinctures." (P. 49.)

After panegyrizing his acid physic, the author attacks the ordinary ways of taking Epsom salts, namely, in plain water, or in infusion of roses, or in infusion of senna; but we cannot afford to quote any more from a short article, and must hasten on to other matter.

Dr. J. SMITH has contributed thirty-six cases of *Insanity*, terminating fatally, with the post-mortem appearances. They show, as the author observes, "that insanity is always connected with some morbid action going on in the brain, its membranes, or the skull, producing eventually, in almost every case, some distinct lesion in one or other of those parts." (P. 384.) These lesions, however, in their turn, become direct causes of insanity; or, in other words, functional leads to organic disease; and, *vice versâ*, where the latter exists, the function of the part cannot be healthily performed. We would suggest to Dr. Smith to throw the principal results of these thirty-six examinations into a tabular form, as it is difficult to read, and impossible to remember, so many cases.

Dr. CRAIGIE'S *Clinical Report* is instructive, but, as it would be in vain to attempt an analysis of more than a hundred closely printed pages, we shall content ourselves with a single quotation.

"*Hydatids.*—The existence of the genuine hydatid is not so frequent as is represented by many pathologists, who have too often, in the case of the ovary especially, confounded with this body common cysts containing limpid serum. Of the genuine hydatid, of the family Cestoidei, one example only occurred this season; but it was highly characteristic and unequivocal. It occurred in the person of Isabella Allan, the patient whom I have already mentioned as exhibiting a perfect specimen of concentric hypertrophy of the left ventricle of the heart, and whom I shall have occasion to mention as an instance of disease of the glandular tissue of the kidney. At the inferior surface of the liver, imbedded in the substance of the left lobe, was a large, rather irregular, elastic tumor, inclosed in an exterior cyst. When this was divided, it was found to contain a quantity of limpid serous fluid; and adherent to its internal surface was a cluster of about seventy or eighty membranous cysts, of a flattened spheroidal shape, with semitransparent coats, and containing limpid serous fluid. These presented the other characters of the echinococcus of Zeder and Rudolphi.

"The convex surface of the liver adhered to the diaphragm firmly and extensively by a considerable quantity of albuminous exudation, effused in columns and shreds, of some standing.

"The peritoneal cavity contained about four pounds of serous fluid. The intestines were much distended by air; and the peritoneal covering adhered in various parts to each other by means of lymph, partly recent, partly of old formation; and these adhesions were most frequent among the pelvic viscera, and on the ascending arch of the colon, the peritoneal covering of which also adhered to the abdominal peritoneum, and to several of the convolutions of the small intestines by lymph of recent formation. On dividing the upper part of the rectum, a considerable quantity of purulent fluid escaped, and was found to proceed from a cyst formed by adhesions between the anterior part of the rectum and the superior and posterior part of the uterus and ligamenta lata.

"The course of the gastro-colic omentum was occupied by a pretty firm albuminous exudation, which gave it considerable thickness." (P. 119.)

Dr. SPITTAL has taken the trouble to go through some of the more noted works on the *Diseases of the Heart*, and has given a table of the cases in which a diagnosis was attempted: this he has done with a view of ascertaining whether auscultation is capable of deciding which side of the heart is diseased. It is hardly necessary to tell our readers that cardiac auscultation is in its infancy, not to say, in an embryo state. It is not yet decided what functions the normal sounds of the heart indicate. How then shall we extricate ourselves from the labyrinth of morbid raspings and gratings? Or which of our readers will be surprised at Dr. Spittal's conclusions?

"It is quite evident, from a perusal of the table, that there is a very great want of agreement in the cases therein mentioned, between the signs afforded by auscultation and the actual condition of the heart; and a careful comparison between the first, second, and fourth columns, will show that a correspondence between the signs afforded and the morbid conditions of the heart, according to the rules laid down by Laennec, occurs only in about one fifth part of the whole; while in a large number the indications are either entirely or in part erroneous, incomplete, or altogether absent: and, to show how little such indications are to be trusted, even in the hands of the eminent individuals whose works we have consulted, it may be mentioned, that of the eighteen cases, each of which is furnished with a diagnosis, only one fourth are, as a whole, correct. With these exceptions, almost all the rest are incomplete, and many are very erroneous.

"We have thus endeavoured to show the actual state of the evidence on the question at issue; and enough, we trust, has been advanced to induce us to pause a little before coming to join in any conclusion analogous to that so decidedly advanced by Laennec, and apparently so cordially supported by those who have followed in his steps. We do not say that such an opinion is in every case erroneous, but that exceptions to it are very numerous; and, from this fact alone, it will in all probability become of little importance; misleading the observer who puts his trust in it, should it even be proved true in some cases. It is quite impossible, however, to come to a satisfactory conclusion at present, and we fear it will remain so until a far more extensive mass of evidence on this subject shall have been accumulated." (P. 148.)

Dr. Macnish gives a *Case of Tumour in the Region of the Liver, with Discharge of Biliary Calculi through the Parietes of the Abdomen, which terminated favourably.* The recovery of the patient was perfect, with the following exception: "For a little space around the cicatrix, which is nearly two inches below the margin of the ribs, the liver is hard, and adherent to the abdominal parietes, and feels uncomfortable on pressure; but no vestige of the pendulous tumour can be discovered." (P. 152.)

Dr. Malcolm relates a case *illustrative of the Spontaneous Evolution of the Fœtus.* When he first saw the patient, an arm, with the shoulder, and a portion of the thorax, had already been expelled through the os externum. Dr. M. administered 150 drops of Tr. Opii, which however increased, instead of weakening, the uterine contractions. He then introduced, with difficulty, two fingers into the vagina, and

"had the good fortune to find both feet, near the foramen externum, jammed together between the lower part of the sacrum and the external opening, in contact with the vagina. From the position of the feet, I judged that the breech was resting at the top of the sacrum, or to one side of it, and the head consequently at the anterior part of the pelvis. I gradually brought the feet forward, and was at length enabled to accomplish the delivery. The feet, breech, body, remaining arm, and lastly the head, being adapted to the pelvis, as in an ordinary footling case, without the hand of the operator being introduced, or the presenting arm returned, into the vagina; in short, without turning having been had recourse to. The perinæum, although much stretched during the delivery, remained entire." (P. 339.)

So that it was a *tertium quid,* between a case of turning and of spontaneous evolution.

Dr. John Davy has an essay on the *Maceration in Water of different Textures of the Human Body.* In an abstract of this kind it is of course impossible to give the author's experiments; it will be enough to quote his deductions from them.

"The general conclusion which I am disposed to draw from these experiments is in opposition to the doctrine of Bichat on the subject of membranes; a doctrine, it must be confessed, which bears the impress of genius; but, I apprehend, rather a fiction of the mind than a well-grounded theory or generalization of facts. In the present state of our knowledge, it appears to me that the doctrines of Haller, on the composition of the textures of the body, are preferable, both in relation to matter of fact and utility of application, to the more modern ones of the French school. It seems to be taken for granted in the new doctrine, that continuity of surface is necessarily accompanied with identity of structure. If the doctrine be correct, this dogma may be admitted; but if the latter is not correct, I do not know how the doctrine itself can maintain its ground. And it appears to me that facts have been advanced in this paper not reconcileable with this dogma. Compare the effects of maceration on the textures of the different passages or canals communicating with the open air; or compare the effects of this process on different parts of the surface of the same canal, and certainly identity of composition is not indicated, but considerable variety. Make the same kind of comparison with different parts of the cutis, and the conclusion is analogous. Far different in composition and in appearance, and in the morbid affections to which they are liable, are the cutis of the sole of the foot, the cutis of the scrotum, of the chin, and of the back." (P. 364.)

Dr. A. Murray proposes to ascertain the presence of minute portions of arsenic, by applying liquid tests to the solid poison, in the following manner:

"1. A small quantity of common arsenic was pressed into writing paper by friction with a glass rod, and upon the stain thus produced a single drop of a solution of common nitrate of silver was rubbed by means of the rod. A phial containing common hartshorn was then immersed in hot water, and over its open mouth the paper was held, (the arsenic being on the upper surface, for the sake of better observing the appearances,) when the whitish spot became in a few seconds lemon yellow. The arsenical spot being kept over the hartshorn for about half a minute more, the yellow colour entirely disappeared, but it returned when the paper was removed from the phial, and held close to the water.

"2. Upon an arsenical stain of the kind described was rubbed a drop of solution of sulphate of copper, and the paper was then held over hartshorn placed as before. Almost immediately a mixture of blue and green streaks was perceived, and in a few seconds more the spot became of a beautiful blue colour. The paper was then removed from the hartshorn, and allowed to float upon the hot water, when the blue in a few seconds was converted into a decided green." (P. 365.)

Mr. John Roberton, surgeon to the Manchester Lying-in Hospital, has contributed some useful *Remarks on the Relaxation and Descent of the Uterus and Bladder in the Puerperal State.* After narrating a number of cases, he says,

"1. Descent of one or more of the pelvic viscera is not, as is commonly believed, a disease chiefly of middle or advanced life. It is true, complete prolapse, the last miserable state of the disease, is generally found in those who have passed their thirty-fifth or fortieth year; but the first stage of descent ordinarily commences early in the childbearing period of life.

"2. In a majority (perhaps I might say in a great majority) of instances, the complaint follows a first labour; and that from causes which have already been stated.

"3. When first labours are tedious, more especially when this appears to be owing, in any measure, to the great size of the child, it behoves us to be on our guard against the occurrence of the disease during the puerperal period,—that is, during the first six weeks." (P. 402.)

Mr. Roberton disapproves of astringent lotions, and recommends those afflicted with this disease in the slightest degree never to suckle longer than five or six months. We agree with the author in the latter point, as nothing can be more certainly destructive of a weak constitution than the long and draining sucklings which we so often see among the poor; but we should reluctantly give up the use of astringent injections, as we believe them to be not only useful, but immediately and powerfully so.

Several other articles contain matter worthy of being transferred to our pages; but we hasten to conclude this notice, lest our readers should find fault with the re-distillation of papers already subjected to an editorial alembic. Yet we would ask, what can be more useful, or more consonant with the character of a practical Journal like this, than the humble office which we have just performed, of analysing and abridging, and giving in a few pages, the pith of volumes?

